# Infection Control Practices Among Private Practicing Dentists in Nairobi During the Pre-coronavirus Disease 2019 Period

**DOI:** 10.3389/froh.2020.587603

**Published:** 2020-12-16

**Authors:** Benedict Odhiambo Otieno, Eunice Njeri Kihara, Bernard Nzioka Mua

**Affiliations:** ^1^Department of Periodontology/Community and Preventive Dentistry, School of Dental Sciences, University of Nairobi, Nairobi, Kenya; ^2^Department of Oral and Maxillofacial Surgery, Oral Pathology and Oral Medicine, School of Dental Sciences, University of Nairobi, Nairobi, Kenya

**Keywords:** infection control, dentists, pre-COVID-19, personal protective equipment, hand hygiene

## Abstract

**Background:** Cross-infection control is a dynamic field that requires frequent updates due to emerging diseases, advancement in technology, and scientific knowledge. Despite wide publication of guidelines, a laxity in compliance to the standard precautions for infection control by dental health-care personnel (DHCP) has been reported globally. Therefore, there is need to review previous shortcomings in order to adequately secure dental practices during the coronavirus disease 2019 (COVID-19) pandemic. The aim of the study was to determine knowledge and infection control practices by dentists in private practices. The study was done a few months before the first COVID-19 case was confirmed in Kenya.

**Materials and Methods:** The study design was a descriptive cross-sectional study that was carried out in selected private dental clinics located in Nairobi. Data were collected using an interviewer-administered questionnaire. Convenience sampling method was utilized, while data were analyzed using SPSS 20.0.0.0.

**Results:** A total of 71 private dentists participated in the study. Their mean age was 38 years with an age range of 27–55 years. Almost all (70, 98.6%) the dentists were able to define cross infection correctly. Majority (62, 87.3%) correctly differentiated between sterilization and disinfection, while 9 (12.7%) had difficulties. Most (68, 95.8%) of the respondents were aware of the standard precautions for cross-infection control. All participants used face masks and gloves. About half of them (38, 54%) practiced hand washing after removal of gloves and 31 (43.7%) before and after wearing of gloves, while 2 (2.8%) washed hands only before wearing gloves. Only 31 (42.3%) and 26 (36.6%) participants reported use of rubber dam isolation and impervious barrier, respectively. All the dentists reported disposal of sharps into especially labeled containers, while about half reported use of disposable suction traps and amalgam separators.

**Conclusion:** The dentists had a good knowledge on various aspects of infection control measures that were studied. Use of basic personal protective equipment was widely practiced. There were irregularities in hand hygiene, use of rubber dam, surface barriers, and waste management. The work highlights that many dentists were unprepared to manage infectious risk during the COVID-19 outbreak, which justified the closure of the dental facilities. Development of strategies to promote adequate and safe practice is highly recommended.

## Introduction

The coronavirus disease 2019 (COVID-19) pandemic has globally affected health-care systems and presented unprecedented challenges in infection control. The field of dentistry has been adversely affected due to the high risk of spread of infection especially through aerosol-generating procedures (AGPs) [1, 2]. During the pre-COVID-19 period, oral health-care workers (HCWs) and dental patients had already been reported to have contracted diseases including *Mycobacterium tuberculosis*, hepatitis B and C virus (HBV and HCV), *Legionella pneumophila*, human immunodeficiency virus, and other microorganisms originating from the oral and respiratory cavities [3–6]. The newly confirmed severe acute respiratory syndrome coronavirus-2 (SARS-CoV-2) [7] has already been reported to have infected dental HCWs at a dental hospital in Wuhan [8].

Cross-contamination inadvertently occurs via intimate interaction with contaminated saliva, blood, secretions, excretions, and air droplets or via unintimate interaction through polluted items, for example, sharp instruments, dental waterline (DWL), and surrounding environment [3, 9–12]. In a dental environment, infection may spread among the dental health-care personnel (DHCP) and the patients; hence, the success of infection control depends on the know-how and practices of the involved individuals in regard to adhering to standard precautions for cross-contamination control.

Globally, government agencies and oral health organizations have regularly outlined recommendations and trainings that are geared toward maintaining a safe clinical operating atmosphere and averting possible spread of health-care-acquired infections among DHCP and their clients. The use of personal protective equipment (PPE) serves as an important nontherapeutic measure in curbing the spread of infections. With the COVID-19 pandemic, the DHCP has to reevaluate and strictly adhere to the recommended PPE when performing AGPs. These include surgical respirators and masks, protective goggles, face shield, gloves, work uniform, surgical gowns, and shoe covers [13–18].

Additionally, sterilization and disinfection play a vital role in decontamination in health-care facilities. However, their success depends on availability and effective use of appropriate sterilizing equipment and disinfectants. The Centers for Disease Control and Prevention (CDC)/Food and Drug Administration (FDA) high-level disinfectants and the United States Environmental Protection Agency (EPA) hospital tuberculocidal disinfectants can destroy a broad spectrum of microorganism and can be used for chemical sterilization of instruments. Regular monitoring of sterility through physical, chemical, and biological indicators is paramount. Further, room decontamination can be achieved through the use of low or intermediate disinfectants or their equivalents. Notably, most viruses including, HIV, HBV, HCV, herpes simplex, and coronavirus are inactivated by the EPA-labeled hospital disinfectants or the CDC/FDA low-level disinfectants [13]. Nevertheless, the conventional methods of room disinfection are operator dependent; hence, adjunct non-touch/automated systems such as UV germicidal irradiation and use of aerosolized hydrogen peroxide have been developed [19–22].

Standard precautions should be undertaken to monitor and maintain clean dental unit waterlines with <500 colony-forming units (CFU) per milliliter as recommended by the CDC [13]. Suggested measures include removal of retracted materials from rotary and ultrasonic instruments as well as air-water syringes, regular disinfection of the suction lines and DWL to remove bacterial biofilm, and use of sterile water to perform procedures [13, 23–25]. The National Guidelines for the Control of Legionellosis in Ireland recommends undertaking of *Legionella* annual assessment on the risk of *Legionella* infection from DWL [26].

Good ventilation in a dental practice is invaluable in protecting from infection by long-range contaminated airborne droplets [10]. When performing AGPs in suspected or confirmed COVID-19 patients, the WHO recommends the use of rooms with negative pressure of a minimum of 12 air changes per hour, while in natural ventilation, it should be at least 160 L per second per patient [27]. In addition, the CDC recommends use of portable high-efficiency particulate air (HEPA) filtration unit [1]. Further infection control precautions include sharps safety, hand and respiratory hygiene, and safe injection practices. Regular training and monitoring the adherence to infection control guidelines are vital [13, 14, 17, 28–30]. Dentists can customize checklists or utilize published audit tools or mobile phone applications to audit their practices regularly [29, 30].

A laxity in the compliance to infection control and preventive measures by some DHCP has been reported all over the world; hence, evaluation of infection control practices and associated factors is worthwhile [23, 31–34]. Furthermore, occasional disease outbreaks and pandemics justify review of routine guidelines as well as issuing updates and promoting stringent adherence in order to protect the DHCP and their patients. The first COVID-19 case was reported in Kenya on 13 March 2020; since then, dentists had to suspend routine dental procedures pending further guidance from Kenya Dental Association, WHO, and FDI [18, 27, 35]. Data on the knowledge and pre-COVID-19 practices of cross-infection control in private dental clinics in Kenya were hardly documented. This study had been carried out just before COVID-19 was reported in Kenya. The aim was to assess the knowledge and infection control practices in private dental clinics in Nairobi. Therefore, it highlights significant issues that require immediate intervention so as to reduce the risk of transmitting the novel coronavirus in a dental setting. The study also reviews the standard precautions for infection control and current recommendations that are aimed at combating the spread of the SARS-CoV-2 and other microorganisms in the dental setting.

## Materials and Methods

The study was carried out in Nairobi County, which is the largest city and the capital of Kenya. It is also a fast-growing city in Africa and is cosmopolitan with a population of 4,397,073 as per the 2019 census. Nairobi County is the administrative and economic center of Kenya, which also offers both public and private health-care services. Majority (208, 60%) of the private dental facilities in Kenya are found in this County. The public dental facilities include nine hospitals and one university dental hospital.

The study design was descriptive cross-sectional study. The study population included both general and specialized private practicing dentists. They were selected through convenience sampling technique from a national list of 344 private dental facilities that were registered by the Kenya Medical Practitioners and Dentists Council (http://kmpdc.go.ke/Registers/H-Facilities.php). The list included the names and postal addresses of the dental practices, but their telephone contacts and physical locations were not indicated. Hence, the study could only include the private clinics, which were readily accessible.

The data were collected by means of an interviewer-administered questionnaire, which was administered in person to the dentists at their private dental clinics. The questionnaire comprised a total of 17 questions, which were distributed in three categories of four, five, and eight questions involving the sociodemographic characteristics, knowledge, and practice in cross-infection control, respectively. Sociodemographic characteristics that were investigated comprised age, gender, professional qualification, and length of time in dental practice. Knowledge-related questions included definition of infection control, sterilization, and disinfection. Practice-based questions inquired about hand hygiene, use of PPE, sterilization and disinfection, waste disposal, and needle-prick injuries. The bulk of the questions were self-generated from the CDC guidelines on standard precautions for infection control in dental settings. Majority had choices so as to control interview bias. The questionnaire was also pretested while the interview was conducted by the first author, who was trained prior to conducting the interviews.

Permission to conduct the study was granted by Kenyatta National Hospital and University of Nairobi, Ethics and Research Committee after a peer review of the research proposal. Permission was granted under approval number UP951/11/2019. Informed consent was sought from the participants before the interview was conducted. Information attained from the study was treated with confidentiality and only for purposes related to the study. Data analysis was done using MS Excel and SPSS version 20.0.0.0 Chi-square test (χ^2^) of association was used to determine the association between the sociodemographic variables and infection control practices. A *p*-value of <0.05 showed statistical significance.

## Results

### Sociodemographic Data

A total of 75 private dentists practicing in Nairobi were approached, and 71 (94.7%) of them consented to participate in the study. Their mean age was 38 years with an age range of 27–55 years and a median age of 36 years. The participants comprised 33 (46.5%) males and 38 (53.5%) females. Majority (56, 78.9%) had only undergraduate training in dentistry, while 15 (21.1%) had postgraduate qualification. The period of practice ranged from 2 to 29 years, while majority (33, 46%) had practiced for 2–10 years. Further information is provided in Table 1.

**Table 1:** Sociodemographic characteristics of the study participants.

**Sociodemographic characteristics**		**Male (%) *n* = 33**	**Female (%) *n* = 38**	**Total (%) *n* = 71**
Age groups (years)	21–30	6 (18)	8 (21)	14 (20)
	31–40	14 (42)	18 (47)	32 (45)
	41–50	11 (33)	6 (16)	17 (24)
	51–60	2 (6)	6 (16)	8 (11)
Time period in dental practice (years)	0–10	15 (45)	18 (47)	33 (46)
	11–20	14 (42)	10 (26)	24 (34)
	21–30	4 (12)	10 (26)	14 (20)
Highest level of dental education	Undergraduate (BDS)	26 (79)	30 (79)	56 (79)
	Masters (MDS)	7 (21)	8 (21)	15 (21)

*BDS, Bachelor of Dental Surgery; MDS, Master of Dental Surgery*.

### Knowledge on Cross Infection

All except one (70, 98.6%) dentist were able to define cross infection correctly. Majority (62, 87.3%) were also able to correctly differentiate between sterilization and disinfection, while 9 (12.7%) had difficulties. Most (68, 95.8%) of the respondents were aware of the standard precautions for cross-infection control. In regard to knowledge on the cross-infection control measures currently available in Kenya, all (71, 100%) were aware about sterilization and disinfection measures, 70 (98.6%) knew about hand hygiene and use of PPE, 69 (97.2%) were aware about the availability of color-coded containers for dental waste disposal, and only 45 (63.4%) were aware of the availability of disposable suction traps and amalgam separators. Majority (56, 78.9%) of the dentists attended continuing professional development workshops on cross-infection control on a yearly basis and 12 (16.9%) every 6 months, while 3 (4.2%) attended on a monthly basis.

### Infection Control Measures Practiced by the Dental Practitioners

In regard to infection control measures practiced by the dentists, about half of them (38, 54%) indicated that they practice hand washing after removal of gloves and 31 (43.7%) before and after wearing of gloves, while 2 (2.8%) of them washed hands only before wearing gloves. The most (70, 98.6%) common method of sterilization was use of moist heat. Chemical sterilization was also utilized by a third (22, 31%) of the dentists, and only two (2.8%) used dry heat. The type of disinfectant used was not assessed. None of the dentists reported use of radiation or gaseous sterilization.

All (71, 100%) the dentists reported use of gloves and face masks for personal protection during routine dental procedures. They also changed gloves and disinfected the dental unit after every patient. Majority (42, 59.2%) of the dentists reported that they wash their work uniform or surgical scrubs daily, while the rest (29, 40.8%) washed them weekly. Most (23, 70%) of the dentists who had practiced for 1–10 years washed daily. There was significant association between washing of work uniform and the length of time in dental practice [χ^2^(2) = 7.049, *p* = 0.029]. Only 31 (42.3%) participants reported use of rubber dam (RD) isolation, while 26 (36.6%) reported that they wrapped difficult-to-clean dental equipment and surfaces with impervious paper, which was changed after every patient. Majority of the dentists who had practiced for 21–30 years used RD (71%) and surface barriers (64%). There was significant association between the number of years the dentists had been practicing and use of surface barriers [χ^2^(2) = 6.803, *p* = 0.033] and RD [χ^2^(2) = 6.819, *p* = 0.033].

In regard to waste management, all (71, 100%) the dentists reported disposal of sharps into a especially labeled container, while incineration of human dental waste was reported by 43 (60.6%) participants. Most participants (62, 87%) used separately labeled containers to store all scrap/old amalgam. About half (36, 50.7%) of the dentists reported use of disposable suction traps and amalgam separators on the dental suction units. More females (36, 95%) than males reported use of separately labeled containers to store all scrap/old amalgam, which was found to be statistically significant [χ^2^(1) = 4.059, *p* = 0.044]. Majority (54, 76.1%) of the dentists described the correct practice of handling needle-prick injuries during their dental practice. Among the 16 (23.9%) who did not provide a description, 11 (15.5%) stated that they had never been pricked, while 6 (8.5%) consulted their nurses for assistance in handling needle-prick injuries (Table 2).

**Table 2:** Infection control practices.

	**Gender**			**Period of time in dental practice**			
	**Male (%) *n* = 33**	**Female (%) *n* = 38**	**Total (%) *n* = 71**	**0–10 (%) *n* = 33**	**11–20 (%) *n* = 24**	**21–30 (%) *n* = 14**	**Total (%) *n* = 71**
**Washing hands before and after wearing gloves**							
Only before	1 (3)	1 (3)	2 (3)	2 (6)	0 (0)	0 (0)	2 (3)
Only after	19 (58)	19 (50)	38 (54)	14 (42)	13 (54)	11 (79)	38 (54)
Before and after	13 (39)	18 (47)	31 (44)	17 (52)	11 (46)	3 (21)	31 (44)
**Use of personal protective equipment**							
Face masks and gloves	33 (100)	38 (100)	71	33 (100)	24 (100)	14 (100)	71
Washing uniform daily	18 (55)	24 (63)	42 (59)	23 (70)	15 (63)	4 (29)	42 (59)
Washing uniform weekly	15 (45)	14 (37)	29 (41)	10 (30)	9 (38)	10 (71)	29 (41)
**Use of barriers**							
Rubber dam	15 (45)	15 (39)	30 (42)	10 (30)	10 (42)	10 (71)	30 (42)
Surface barriers	15 (45)	11 (29)	26 (37)	8 (24)	9 (38)	9 (64)	26 (37)
**Waste management**							
Incineration	22 (67)	21 (58)	43 (61)	17 (52)	15 (63)	11 (79)	43 (61)
Sharps disposal in special containers	33 (100)	38 (100)	71 (100)	33 (100)	24 (100)	14 (100)	71 (100)
Use of containers for amalgam disposal	26 (79)	36 (95)	62 (87)	29 (88)	22 (92)	11 (79)	62 (87)
Use of amalgam suction traps/separators	18 (55)	18 (47)	36 (51)	13 (39)	12 (50)	11 (79)	36 (63)
Correct handling of needle-prick injuries	22 (67)	32 (84)	54 (76)	27 (82)	19 (79)	8 (57)	54 (76)

## Discussion

Generally, most dentists were found to have a good knowledge in various aspects of infection control that were studied. This is expected, since infection control measures are well studied and fairly practiced during training in dental schools in Kenya. Similar results were observed in a study done at a local university dental hospital [36]. Nevertheless, infection control is a dynamic field that requires regular reviews owing to emerging diseases, technology, and scientific knowledge. This underscores the importance of involvement in continuous education. Most participants were found to attend professional training only once in a year. Dentists can improve their knowledge on infection control by engaging in various online training courses provided by professional societies or government agencies. Irregularities associated with compliance to various infection control measures were noted in our study. This was a great concern especially in the era of COVID-19, which presents a new knowledge gap that requires prompt intervention, research, retraining, and setting up of distinct guidelines in order to prevent the spread of coronavirus in dental settings. Standard precautions have to be strictly adhered to, since asymptomatic/preasymptomatic patients have been found to spread the SARS-CoV-2 [37, 38].

### Hand Hygiene Practices

Hand hygiene plays a crucial role in the prevention of nosocomial infections [13, 39]. It was observed that hand washing before and after changing the gloves was not exclusively practiced in our study, although the alternative use of alcohol-based hand rubs was not investigated. Nevertheless, studies that assessed both hand washing and use of hand rubs still found clinicians who did not comply with the recommended hand hygiene guidelines [33, 40, 41]. A study at a university hospital in Taiwan found that 71.4 and 71.6% of dentists did not adhere to hand hygiene before and after treating patients, respectively [40]. Mutters et al. also observed that majority (64.8%) of dentists did not comply with proper hand hygiene before and after dental procedures [33]. However, Hübner et al. observed an improved behavior in hand hygiene following the implementation of the guidelines by the Commission of Hospital Hygiene and Infection Prevention at the Robert Koch-Institute in 2006 [41]. Hence, frequent training and monitoring are key to improve hand hygiene. The use of alcohol-based hand rubs has also revolutionized hand hygiene and is recommended when the hands are not visibly soiled [42, 43]. Deficiencies in hand hygiene have been noted in the following areas: frequency of hand washing, use of water faucets without touching with the hands, provision, and dispensing and handling of hand care products such as soap, disinfectant hand rubs, and moisturizers [33, 40, 41]. An investigation into factors related to compliance with hand hygiene is highly recommended. Clinicians should also endeavor to strictly adhere to published guidelines on various hand hygiene practices during surgical and nonsurgical procedures [13, 17, 42, 43].

### Use of Face Masks

Face masks were used by all the dentists, and a similar observation was made in a German university. Less compliance was noted by Hübner et al. and Xu et al., where 15 and 52.2%, respectively, did not use surgical masks [5, 41]. Mutter et al. and Sabaya et al. noted that irregularities associated with wearing of face masks included failure to cover the nose and laxity in changing the mask after every patient [33, 36]. According to infection control guidelines, dental health workers should use surgical masks that are impermeable and efficient in protection from microorganisms. During the SARS-CoV-2 pandemic, the filtering face piece respirators such as N95 (United States NIOSH-42CFR84), FFP2 or FFP3 (Europe EN 149-2001), KN95 (China GB2626-2006), and equivalents are recommended on account of their tight fit and superior filtration (>95%) of small airborne droplets (1-μm particles). The far superior EU FFP3 mask is recommended during emergency care of confirmed or suspected COVID-19 patients [13, 44, 45]. Normally, the respirators are discarded after performing AGPs. However, use of respirators is confounded by high cost as well as limited supply; hence, single usage may not be feasible especially during infection-related pandemics, various recommendations on extended use, mask rotation, and/or decontamination after performing non-AGPs have been suggested [44]. Concomitant use of a face shield reduces the contamination of the face and the respirator. The moist heat sterilization, UV irradiation, and hydrogen peroxide fumigation are currently the most favorable methods of decontamination. However, decontamination can be limited due to unavailability of decontaminating systems, degradation of the filter, failure to fit, and odor and toxicity from decontaminants rendering, them unsafe to the user. Health facilities need to establish their own practical evidence-based standard operating procedures on use and reuse of respirators based on available resources [1, 46–54]. A research on the current trend in facial protection in dental health-care facilities during the COVID-19 outbreak is highly recommended.

### Use of Health-Care Personnel Attire

The majority of the dentists in Nairobi used work uniform (laboratory coats/surgical scrubs) that was cleaned daily. A lower frequency was reported at a dental hospital in India where majority of clinicians washed their white coats once a week [55]. Studies have found that white coats worn in dental hospitals become colonized majorly by gram-positive microorganisms [55, 56]. *Staphylococcus aureus* has been the commonest microorganism isolated from white coats and other uniforms worn by HCWs [57–60]. However, there is paucity of data on the spread of infections to patients through attire worn by HCWs. The standard guidelines for infection control recommend change of protective clothing as soon as they are soiled. The DHCP should also refer to local and universal guidelines on management of soiled laundry. Nonetheless, washing at high temperatures, use of hypochlorite, and ironing allow elimination of the microorganisms [13, 17, 61, 62]. Due to the SARS-CoV-2, the Occupational Safety and Health Administration [15] recommends use of gowns when performing AGPS and when treating suspected or confirmed cases of COVID-19, while the CDC also provides additional guidance on how to optimize their supply [15, 53].

### Rubber Dam Isolation

Working in the oral cavity presents a risk of exposure to respiratory pathogens, contaminated blood, and saliva; hence, RD isolation is inevitable [33, 63, 64]. Our study found that a majority of the dentists did not practice under RD isolation. In Germany, a study by Mutters et al. [33] found that only 22.8% respondents used RD. Similar observations were made by Madarati [64] and Gilbert et al. [65], who noted that 62.7 and 53% of their respondents respectively did not utilize RD during endodontics procedures. Our study found the lowest utilization among dentists who had not practiced for long. Further studies to investigate the reasons for the observation are recommended. However, other studies have attributed low usage to various factors including inadequate training, failure to invest in RD isolating materials, time-consuming process for the inexperienced clinicians, and negative attitude toward the effectiveness of RD [63–65]. Training on RD isolation should focus on improving skills and attitude toward its use. Besides RD isolation, preoperative gargling with an antimicrobial provides further protection, and povidone-iodine has been found to be effective especially against coronaviruses such as SARS-CoV and Middle East respiratory syndrome (MERS)-CoV [66, 67]. Further studies to investigate virucidal effects of available mouthwashes against the SARS-CoV-2 are ongoing.

### Sterilization and Disinfection

In the current study, moist heat was the most (98.6%) popular method of sterilization. Similar results were found at a different local study where 93.1% used moist heat, while about a third (34.5%) also utilized chemical sterilization [68]. In contrast, a study by Yuzbasioglu et al. [32] among Turkish dentists showed that majority (65.9%) had sterilization facilities for dry heat, while 46.7% had autoclaves and 34.8% used chemical sterilization. Moist heat sterilization is normally the most common method among the dentists, while the dry heat method is only suitable for instruments that can withstand high temperatures of 170°C. In general, instruments classified as critical require sterilization or single use since they come into contact with sterile tissues, while the semicritical ones can be sterilized or disinfected with high-level disinfectant [19–21, 69]. Monitoring of the effectiveness of sterilization is recommended; a previous local study found sterilization failure in 31% of autoclaves that were monitored through biological indicators [68].

It is worth noting that all the clinicians disinfected the dental chair after every patient, though the type of disinfectant used was not reported. A review of literature showed that previously known coronaviruses persisted on surfaces for up to 9 days, but surface disinfection with 0.1% sodium hypochlorite or 62–71% ethanol inactivated them [70]. In our study, it was noted that only few dentists used disposable barrier covering despite the fact that they have been found to be economical and provide further protection in frequently touched surfaces, equipment, and dental material dispensers. To this end, impervious materials are recommended as barrier protection for surfaces and equipment that are difficult to decontaminate; hence, cleaning can be done at the end of the day. New barrier covering is placed between patients, while soiled surfaces require immediate decontamination [13, 24, 71, 72].

### Waste Management

Proper handling and disposal of clinical waste play a critical role in controlling the spread of diseases and harm to the public. In our study, incineration of human dental waste was done by a majority of the dentists. A local study done by Osamong et al. [73] found that 52.4% of the dentist incinerated pathological waste, while 28.6% disposed of it as general waste. The Infection Prevention and Control guidelines for health-care services in Kenya recommends the incineration of infectious waste [17]. Further studies should be undertaken to assess the level of knowledge and adherence to local policies on waste management.

Majority of the sampled practices kept amalgam in separate containers, but only half of them had installed amalgam separators, indicating that amalgam was still ending up in the sewerage system. The local dental institutions are expected to support the efforts of the amalgam phase-down project in Kenya, which had already installed separators at three dental institutions as a demonstration of safe management of amalgam waste [74] The International Academy of Oral Medicine and Toxicology (IAOMT) recommends the use of amalgam separators as part of Safe Mercury Amalgam Removal Technique (SMART) [75]. Moreover, in 2017, EPA ruled that all the dental units that place or remove amalgam must be installed with amalgam separators by 2020 July [76]. Globally, there is need for continuous monitoring on management of amalgam waste in order to promote protection from the harmful effects of mercury.

Segregation of sharps into especially labeled containers was practiced by all dental practices. A study by Mutters et al. [27] also found majority (96.3%) of the dental practices to dispose the sharps safely. In our study, most dentists were aware of the correct procedure followed in the case of a needle-prick injury during their dental practice. This is in contrast to a study by Mungure et al. [28], which observed that only 27% of under- and postgraduate students at a local university dental hospital knew the procedure undertaken in case of a needle-prick injury. The few dentists who were not familiar with the correct way of handling needle-prick injuries are still of concern. Hence, there is a need for further training and development of a customized protocol for sharps safety. Our study could be limited due to response bias, while not all aspects of infection prevention and control were assessed. The results cannot be generalized since only a convenience sample of both generalized and specialized dentists practicing in Nairobi was used. In addition, the investigated infection control practices were not specific to any dental procedure. It is possible that dentists could have different practices for surgical and nonsurgical procedures.

## Conclusion

The dentists had a good knowledge on various aspects of infection control measures that were studied, and basic PPE was generally used. However, there were irregularities in hand hygiene, use of RD, surface barriers, waste disposal, and sharps safety. The work highlights that many dentists were unprepared to manage infectious risk during the COVID-19 outbreak, which justified the closure of the dental facilities. Dentists need to reevaluate all aspects of infection control and develop strategies aimed at a more adequate and safe practice. Prompt implementation of necessary standard precautions is paramount as well as keeping up to date with emerging challenges and special precautions associated with prevention of the spread of SARS-CoV-2.

## Data Availability Statement

The raw data supporting the conclusions of this article will be made available by the authors, without undue reservation.

## Ethics Statement

The studies involving human participants were reviewed and approved by Kenyatta National Hospital and University of Nairobi Ethics and Research Committee. The patients/participants provided their written informed consent to participate in this study.

## Author Contributions

BO collected data while BM and EK monitored the process. BO and EK analyzed the data. EK wrote the manuscript, which was approved by all authors. All authors participated in writing the protocol for ethical approval.

## Conflict of Interest

The authors declare that the research was conducted in the absence of any commercial or financial relationships that could be construed as a potential conflict of interest.
